# Correspondence

**Published:** 1897-08

**Authors:** 


### CORRESPONDENCE.

EDITORS JOURNAL OF COMPARATIVE MEDICINE, ETC.:

In a review of the translation of Müller's work on the dog I was surprised to find the following published in the June number of your JOURNAL, from one who is supposed to understand what is due to the fellow-members of his profession: "It is true that a number of books upon diseases of the dog have been published, notably those of Hill, Steel, and Mills; but these works are not of a thoroughly scientific character. They are written for the layman as well as the veterinarian, and are a little more than compilations of the popular knowledge of these matters."

A good cause and a good book can stand on their merits, and do not require the belittlement of the works of others; but when authors so treated are *named*, it is perfectly plain that well-understood principles of professional ethics are violated just as much as if one practitioner were to write in the JOURNAL: "Do not employ Dr. ——, for he is a shallow fellow with very little knowledge of what he professes; but go to Dr.——, who is really the only man in the county who knows thoroughly what he is about." Moreover, the statement above quoted from the "review" in question is, so far as my own work is concerned, not correct, and I am prepared to challenge the reviewer to show that my work on the dog is either "not of a thoroughly scientific character," or "little more than a compilation of the popular knowledge of these matters."

**Canada and Manitoba will be represented at Nashville.**

When I set about writing my book, in 1891, I realized that in the larger proportion of veterinary colleges no specific course was given on the dog; that equine medicine is not canine medicine; that while the larger number of students of veterinary medicine are fairly well acquainted with the various farm animals in health, such knowledge of the dog is very rare among them; that unfortunately too large a proportion of students of comparative medicine read regularly no books bearing on their profession except some works on equine anatomy, relying on lectures and the notes taken while listening to them for their entire knowledge; hence, I was determined, in the limitations and character of the book I was to write, by the facts of the case as they exist in America, not in

Great Britain or in Germany. What proportion of the students of American veterinary colleges read any work on the dog whatever? What likelihood is there that they will peruse a large and elaborate and expensive work? We must creep before we walk.

**Control work and sanitary science will have strong and able devotees at Nashville.**

I endeavored to produce a work that would treat of the dog in health and in disease, which would be scientific, yet not beyond the student and the busy practitioner; but while scientific I realize that it must not bristle too much with technicalities nor aim to be exhaustive. The fact that soon after its publication it was recommended in the prospectuses of most of the colleges, and that in three years a second edition was called for, would seem to indicate that in the opinion of the profession it had served a good purpose and met a real need.

Müller's work on the dog is a very valuable work, translated by an able man; but just because it is so, it does not stand in need of methods of pushing which violate professional ethics and are open to legal action. I am glad such a work is accessible to the practitioner to peruse in his own language, and to the more ambitious undergraduate to consult; but to many it will prove strong meat. However, when the time comes that the great mass of students and practitioners regularly read and digest such works, none will rejoice more than myself at the progress made. In the meantime the law of "survival of the fittest" may be left to work itself out in regard to works on canine medicine without deliberate violations of other equal if not more important laws.

Your, truly, WESLEY MILLS.

McGill University, Montreal, June 23, 1897.

EDITORS JOURNAL OF COMPARATIVE MEDICINE, ETC.:

As I am responsible for the review of Dr. Glass's translation of Müller's work on *Diseases of the Dog*, to one portion of which Prof. Mills has taken exception, I hope that you will permit me to say a word in reference to the sentence quoted by Dr. Mills and his criticism upon it.

Without going into the matter in greater detail, it is sufficient to say that Prof. Mills objects to having his work classed with those of Hill and Steel, and the statement that these books are not of a thoroughly scientific character. A careful perusal of Prof. Mills's letter will show that he himself strongly supports this position, although he affects to take exception to it. He admits that his book is an elementary, popular work, and is not put forth as an exhaustive and scientific treatise on the diseases of the dog. Why, then, should I occupy more space in still further refuting an objection which Prof. Mills makes and then so effectually disproves?

The statements that are made in reference to the works of Messrs. Hill, Steel, and Mills are not to their discredit. The books written by them are useful. There is a demand for them, and it is proper that this demand should be met. These works have done much to create a demand for a book of a higher and more scientific character—a book that deals with canine pathology in a more exhaustive manner, and it is this demand that has been recognized and satisfied by Dr. Glass.

There is no need of entering into a controversy with Prof. Mills in regard to professional ethics. I should be very sorry to have written anything that could be regarded as the slightest breach of the strictest code. But what must be the opinion of one who reads the clumsily disguised allusions and the baseless insinuations in the letter of Prof. Mills? I have always believed Prof. Mills to be a thoroughly upright scientist, and one who was deeply interested in the progress of veterinary medicine and veterinary education, and in the present case I regard it as more charitable to think that Prof. Mills is seeking a free advertisement for his own book than to consider his letter an attack upon a work that seems to infringe in a field that he has endeavored to occupy.

Yours, truly, LEONARD PEARSON.

Philadelphia, Pa., July, 1897.

### ENCOURAGING THE IGNORANCE OF RABIES.

EDITORS JOURNAL OF COMPARATIVE MEDICINE, ETC.:

In the May issue of the *American Veterinary Review* a veterinarian in Brooklyn reported six cases of dumb rabies occurring in two weeks. Is this not remarkable, when we take into consideration the fact that this is one of the rarest of diseases?

**Will the Association change its name to American Veterinary Association?**

There are many neuroses in the dog which may simulate rabies, and a mere paralysis of the masticatory muscles is no indication or pathognomonic of rabies. I have had two cases of paralysis of the lower jaw, one caused from a blow on the head, the other with protruding tongue, with partial paralysis from swallowing a rag. We have many neuroses in the dog due to reflex irritation.

The American Society for the Prevention of Cruelty to Animals declare that during the thirty years of their existence there has been no single well-established case of rabies or hydrophobia. This was confirmed by the experience of the most eminent physicians.

Is there an eminent neurologist in the city of New York who has seen a case in his practice?

In the July issue of the *Review*, under the heading "Encouraging the Spread of Rabies," the author, after recording a case in Brooklyn directly traceable to the bite of a dog, which was sold from the shelter, in which he says the animal gave pathognomonic symptoms of dumb rabies, goes on to say that "it appears to us that any animal not claimed by an owner should be put to death, as they have been exposed (as it has been exposed) to too much danger to form a proper companion of human beings (for) (note the grammatical errors) thereby, that we must deny even the owner from redeeming his dog, for would he not be exposed to the danger of hydrophobia also?''

**Editor Bell of the "Review" shows a keen journalistic interest in the success of the Nashville Meeting.**

What are the pathognomonic signs of rabies? Dr. Charles W. Dulles, the eminent lecturer on the history of medicine at the University of Pennsylvania, after sixteen years of investigation has failed to find a single case on record that could be conclusively proved to have resulted from the bite of a dog. The so-called cases of hydrophobia have been produced by a morbid imagination simulating the disease, and tetanus and septic infection have been mistaken for it when accompanied by the hallucinations of a disordered mind. Then let rabies and hydrophobia, until such diseases are conclusively proved, be relegated to the abandoned vagaries, assure the public mind that it is only imagination, and we will see very little of it.

R. B. Plageman, D.V.S.

1432 Bedford Ave., Brooklyn, July 16, 1897.

---

### PENNSYLVANIA STATE BOARD OF VETERINARY MEDICAL EXAMINERS.

Editors Journal of Comparative Medicine, etc.

Veterinarians desire to inquire if the practice of meat- and milk-inspection by laymen officials appointed by local authorities in several cities in this Commonwealth is in violation of the present existing veterinary law? Also, would it be a violation of law or a duly appointed veterinary inspector in the Bureau of Animal Industry, U. S. Department of Agriculture, to engage in the performance of his regular duties in any part of Pennsylvania without first having secured a license or its equivalent from the above-named board? It might be business policy to have this matter brought in proper form before the Attorney-General.

There is another point that is not fully understood yet, viz.: Veterinary college graduates who had practised in Pennsylvania before enactment of any veterinary laws, then entered the government service in legal form, and on duty in other States, or had moved away, or retired from general practice, might desire to return and resume practice, but are not aware whether they are eligible, or will be required to pass the usual examination. These matters ought to be decided by the proper authority, then give us the information through the usual channels and THE JOURNAL OF COMPARATIVE MEDICINE AND VETERINARY ARCHIVES.

Respectfully submitted for official action,

JAMES A. WAUGH.

Pittsburg, Pa., July 25, 1897.

